# Genetic variation in *PRL *and *PRLR*, and relationships with serum prolactin levels and breast cancer risk: results from a population-based case-control study in Poland

**DOI:** 10.1186/bcr2864

**Published:** 2011-04-06

**Authors:** Sarah J Nyante, Jessica M Faupel-Badger, Mark E Sherman, Ruth M Pfeiffer, Mia M Gaudet, Roni T Falk, Abegail A Andaya, Jolanta Lissowska, Louise A Brinton, Beata Peplonska, Barbara K Vonderhaar, Stephen Chanock, Montserrat Garcia-Closas, Jonine D Figueroa

**Affiliations:** 1 Division of Cancer Epidemiology and Genetics, National Cancer Institute, 6120 Executive Boulevard, Rockville, MD 20852, USA; 2 Cancer Prevention Fellowship Program, Center for Cancer Training, National Cancer Institute, 6120 Executive Boulevard, Rockville, MD 20852, USA; 3 Epidemiology Research Program, American Cancer Society, 250 Williams Street, Atlanta, GA 30316, USA; 4 Department of Cancer Epidemiology and Prevention, Cancer Center and M. Sklodowska-Curie Institute of Oncology, WK Roentgena 5, 02-781 Warsaw, Poland; 5 Department of Occupational and Environmental Epidemiology, Nofer Institute of Occupational Medicine, Teresy 8 St., Łódź 91-348, Poland; 6 Mammary Biology and Tumorigenesis Laboratory, Center for Cancer Research, National Cancer Institute, Building 37, Bethesda, MD 20892, USA; 7 Division of Cancer Epidemiology and Genetics, National Cancer Institute, 8717 Grovemont Circle, Gaithersburg, MD 20852, USA; 8 Institute of Cancer Research, 15 Cotswold Road, Belmont, Sutton, Surrey, SM2 5NG, UK

## Abstract

**Introduction:**

Studies suggest that high circulating levels of prolactin increase breast cancer risk. It is unclear if genetic variations in prolactin (*PRL*) or prolactin receptor (*PRLR*) genes also play a role. Thus, we examined the relationship between single nucleotide polymorphisms (SNPs) in *PRL *and *PRLR*, serum prolactin levels and breast cancer risk in a population-based case-control study.

**Methods:**

We genotyped 8 *PRL *and 20 *PRLR *tag SNPs in 1965 breast cancer cases and 2229 matched controls, aged 20-74, and living in Warsaw or Łódź, Poland. Serum prolactin levels were measured by immunoassay in a subset of 773 controls. Odds ratios (ORs) and 95% confidence intervals (CIs) for genotype associations with breast cancer risk were estimated using unconditional logistic regression, adjusted for age and study site. Geometric mean prolactin levels were estimated using linear regression models adjusted for age, study site, blood collection time, and menstrual cycle day (premenopausal women).

**Results:**

Three SNPs were associated with breast cancer risk: in premenopausal women, *PRLR *rs249537 (T vs. C per-allele OR 1.39, 95% CI 1.07 - 1.80, *P *= 0.01); and in postmenopausal women, *PRLR *rs7718468 (C vs. T per-allele OR 1.16, 95% CI 1.03 - 1.30, *P *= 0.01) and *PRLR *rs13436213 (A vs. G per-allele OR 1.13 95% CI 1.01 - 1.26, *P *= 0.04). However, mean serum prolactin levels for these SNPs did not vary by genotype (*P*-trend > 0.05). Other SNPs were associated with serum prolactin levels: *PRLR *rs62355518 (*P*-trend = 0.01), *PRLR *rs10941235 (*P*-trend = 0.01), *PRLR *rs1610218 (*P*-trend = 0.01), *PRLR *rs34024951 (*P*-trend = 0.02), and *PRLR *rs9292575 (*P*-trend = 0.03) in premenopausal controls and *PRL *rs849872 (*P*-trend = 0.01) in postmenopausal controls.

**Conclusions:**

Our data provide limited support for an association between common variations in *PRLR *and breast cancer risk. Altered serum prolactin levels were not associated with breast cancer risk-associated variants, suggesting that common genetic variation is not a strong predictor of prolactin-associated breast cancer risk in this population.

## Introduction

The reproductive hormone prolactin is produced primarily by the pituitary gland and in lesser amounts by several other tissues, including breast tissue. Prolactin plays a central role in breast development, differentiation, and lactation (reviewed in [1]), but experimental data suggest that, in addition to having a role in normal development, prolactin may have procarcinogenic effects (reviewed in [2,3]). Prolactin and prolactin receptors are present in normal breast tissue, benign breast disease, breast cancer cell lines, and breast tumor tissue [3-7], leading to speculation that the proliferative and antiapoptotic effects of prolactin in breast epithelial cells could be a factor in breast carcinogenesis [2,8].

There is a growing body of epidemiologic evidence supporting an association between circulating prolactin levels and breast cancer risk, although data are not conclusive. Large prospective studies have reported a positive association between prolactin levels and breast cancer risk [9-11]. No association was reported by smaller prospective studies, although the number of breast cancer cases was limited [12,13]. Results from case-control studies are inconsistent (reviewed in [14]). We recently reported that higher prolactin levels were associated with increased breast cancer risk in our population-based case-control study conducted in Poland [15], and this is consistent with larger cohort studies. We also found that increased serum prolactin levels were associated with nulliparity in premenopausal women and with current or recent use of hormone therapy (HT) and lower body mass indices (BMIs) in postmenopausal women [15].

In addition to reproductive and environmental factors, genetic variation in the prolactin (*PRL*) and prolactin receptor (*PRLR*) genes may be associated with increased prolactin levels and breast cancer risk. Exploration of genetic variants in *PRL *and *PRLR *has identified single-nucleotide polymorphisms (SNPs) that alter transcription factor binding [16], modify prolactin receptor activity [17,18], and may be associated with breast cancer risk [19,20] or circulating prolactin levels [19]. To follow up our previous finding of a relationship between serum prolactin and breast cancer risk [15], we investigated the association between common genetic variation in *PRL *and *PRLR *and breast cancer risk in the Polish Breast Cancer Study. Additionally, we examined the association between *PRL *and *PRLR *SNPs and serum prolactin levels among controls, hypothesizing that altered serum prolactin levels may be an intermediate marker between genetic variation in *PRL *and *PRLR *and breast cancer risk.

## Materials and methods

### Study population

The design of the Polish Breast Cancer Study has been reported [15,21]. Briefly, eligible cases included women (age range of 20 to 74 years) who were living in the cities of Łódź or Warsaw, Poland, and whose primary invasive or *in situ *breast cancer was diagnosed between 2000 and 2003. Cases were identified by means of a rapid identification system at area hospitals, and the Warsaw Cancer Registry was used to identify any additional cases that were missed by the rapid identification system. Eligible controls were identified from a population registry containing demographic information for all residents of Poland and were frequency-matched to cases on the basis of age (5-year categories) and study site. All participants provided informed consent, and the study protocol was approved by ethics committees in Poland and the US. A total of 2,386 cases (79% of eligible) and 2,502 controls (69% of eligible) were enrolled in the study.

Study participants provided information on demographics, reproductive and medical history, oral contraceptive and postmenopausal hormone use, and other potential breast cancer risk factors during a personal interview. Height, weight, and waist and hip circumferences were measured by a trained nurse. A blood sample was provided by 84% of cases and 92% of controls.

### Genotyping

Tag SNPs in *PRL *and *PRLR *were genotyped by using the Fluidigm dynamic array at the National Cancer Institute (NCI) Core Genotyping Facility (Frederick, MD, USA) [22]. SNPs were selected by using genotype data from the International HapMap Project CEU (Utah residents with Northern and Western European ancestry from the CEPH (Centre d'Etude du Polymorphisme Humain) collection) population. SNPs with a minimum minor allele frequency (MAF) of 0.05 were chosen to cover the genes with a minimum pairwise correlation (*r^2^*) of 0.80. Tag SNP selection was performed by using Tagger in Haploview (Broad Institute, Cambridge, MA, USA). SNPs previously reported by Lee and colleagues [19] to be associated with serum prolactin levels or breast cancer risk were also genotyped and were used as tag SNPs where possible. Thirty-four tag SNPs were selected for study; six were excluded because of assay design issues. Four thousand two hundred twenty-one participants had adequate DNA available for Fluidigm genotyping, and genotype calls were made for 3,849 (91%).

Two SNPs (rs2244502 and rs4425481) failed the Fluidigm genotyping and were repeated by using Taqman assays. Four thousand two hundred twenty-nine participants had adequate DNA for Taqman genotyping, and genotype calls were made for 4,047 (96%).

A total of 28 tag SNPs - 8 *PRL *and 20 *PRLR *- were genotyped in 1,965 cases and 2,229 controls, whose characteristics are displayed in Table 1. *PRL *SNP rs9466314 was monomorphic and excluded from further analysis. Genotype frequencies for two *PRLR *SNPs were not consistent with Hardy-Weinberg equilibrium (HWE) in controls (defined as *P *< 0.001: rs7731880 and rs12153280) and these SNPs were also excluded from the analysis. Associations with serum prolactin and breast cancer risk were examined for the remaining 25 SNPs. Linkage disequilibrium (LD) among control subjects was limited; the mean *r^2^*values were 0.21 (range of 0 to 0.84) among sequential *PRL *SNPs and 0.13 (range of 0 to 0.45) among *PRLR *SNPs. Additional positional information and allele frequencies are presented in Table 2.

**Table 1:** Characteristics of Polish Breast Cancer Study participants (*n *= 4,194)

Characteristic	Case		Control		*P *value^a^
		
	Number	Percentage	Number	Percentage	
Age					
Less than 40 years	80	4	92	4	
40-49 years	501	26	554	25	
50-59 years	668	34	759	34	
60-69 years	508	26	588	26	
At least 70 years	208	11	236	11	
Menopausal status					
Premenopausal	521	27	686	31	<0.01
Postmenopausal	1,444	73	1,543	69	
Parity					
Nulliparous	283	14	248	11	<0.01
Parous	1,682	86	1,981	89	
Breastfeeding^b^					
0 months	353	21	368	19	0.03
Less than 12 months	939	56	1,078	54	
12 to 23 months	255	15	336	17	
At least 24 months	135	8	199	10	
Age at menarche					
Not more than 12 years	562	29	497	23	<0.01
13 years	427	22	516	23	
14 years	520	27	615	28	
15 years	208	11	253	11	
At least 16 years	233	12	322	15	
Body mass index					
Less than 25 kg/m^2^	732	37	705	32	<0.01
25 to less than 30 kg/m^2^	696	35	812	36	
At least 30 kg/m^2^	535	27	709	32	
Oral HT use^c^					
Never	1,106	82	1,284	88	<0.01
Current	120	9	68	5	
Recent	64	5	25	2	
Past	62	5	83	6	
Alcohol drinks per week					
Never drinker	1,254	65	1,464	67	0.11
0 to less than 1	184	10	206	9	
1 to less than 3	282	15	289	13	
3 to less than 5	71	4	97	4	
At least 5	137	7	121	6	
Family history of breast cancer^d^					
No	1,766	90	2,101	94	<0.01
Yes	198	10	128	6	

^a^Pearson chi-square test *P *value. ^b^Parous women only. ^c^HT, hormone therapy, postmenopausal women only. ^d^Mother, sister, daughter, excludes half-sisters.

**Table 2:** *PRL *and *PRLR *single-nucleotide polymorphisms genotyped in the Polish Breast Cancer Study

Single-nucleotide polymorphism	Chromosome	Base pair coordinate^a^	Location	Alleles	MAF^b^	*P*_HWE_^c^
*PRLR*						
rs37364	5	35108137	Intron 7	A/C	0.21	0.58
rs43215	5	35122726	Intron 3	G/A	0.12	0.67
rs249537	5	35125057	Intron 3	C/T	0.11	3.0 × 10^-3^
rs7734558	5	35150368	Intron 3	C/T	0.48	0.03
rs62355518	5	35218778	Intron 3	A/G	0.16	0.68
rs10941235	5	35221337	Intron 3	G/A	0.27	0.49
rs13436213	5	35221583	Intron 3	G/A	0.35	1
rs4425481	5	35221654	Intron 3	G/T	0.06	0.20
rs1610218	5	35222451	Intron 3	G/A	0.04	0.63
rs17249539	5	35233127	Intron 3	C/G	0.12	0.79
rs12153280	5	35235019	Intron 3	G/T	0.11	<1.0 × 10^-4^
rs873456	5	35235414	Intron 3	G/T	0.34	0.90
rs7718468	5	35236793	Intron 3	T/C	0.32	0.53
rs34024951	5	35246079	Intron 3	G/A	0.10	0.95
rs9292575	5	35250262	Intron 3	C/A	0.09	0.74
rs10038062	5	35256276	Intron 3	A/G	0.09	0.76
rs7731880	5	35257203	Intron 3	G/C	0.12	<1.0 × 10^-4^
rs931741	5	35257808	Intron 3	C/G	0.31	0.83
rs7735260	5	35262772	Intron 3	C/T	0.16	4.9 × 10^-3^
rs10068521	5	35266137	Exon 1	C/G	0.03	0.42
*PRL*						
rs849872	6	22391011	3' UTR	A/G	0.15	0.60
rs849870	6	22391864	3' UTR	G/A	0.14	0.56
rs1205960	6	22396139	3' UTR	G/A	0.25	0.13
rs849886	6	22399346	Intron 3	G/A	0.46	0.94
rs2244502	6	22402966	Intron 3	A/T	0.31	0.23
rs12202764	6	22403312	Intron 3	A/T	0.27	0.16
rs3756824	6	22406716	5'	G/C	0.03	0.07
rs9466314	6	22414284	5'	T/T	0	1

^a^Single-Nucleotide Polymorphism database (dbSNP) build 36.3. ^b^Minor allele frequency in controls only. ^c^Test of Hardy-Weinberg equilibrium in controls only. *PRL*, prolactin (gene); *PRLR*, prolactin receptor (gene); UTR, untranslated region.

SNP genotyping completion proportions ranged from 88% to 98%. To confirm the accuracy of genotype calls, 152 samples were genotyped in duplicate. Concordance between duplicate samples was less than 98% for three SNPs - rs62355518 (97%), rs7734558 (96%), and rs7735260 (82%) - and at least 98% for the remaining SNPs.

### Serum prolactin measurement

Serum prolactin concentration was measured in a subset of cases and controls, whose selection was described in detail by Faupel-Badger and colleagues [15]. Controls were matched to cases by menopausal status, age (5-year categories), time of blood collection (within 2 hours), study site, and day of menstrual cycle (within 2 days, premenopausal women only). Only control prolactin concentrations were used in this analysis.

Serum prolactin levels were measured by Quest Diagnostics (San Juan Capistrano, CA, USA) by using the Bayer ADVIA Centaur immunoassay (Bayer HealthCare, Tarrytown, NY, USA) and were calculated after calibration with known prolactin concentrations. One control subject was excluded from further serum prolactin analyses because of a concentration outside the assay limits of detection (0.3 to 200 ng/mL). The within-batch coefficient of variation (CV) was 3.84% and the between-batch CV was 2.31%, as reported previously [15].

### Covariates

Age was defined as age at diagnosis for cases or as age at interview for controls and was included in models using 5-year categories. Women were classified as premenopausal if they reported that they were still having natural menstrual periods; medical records were used to determine menopausal status for women who did not know whether they were still experiencing natural menstrual periods.

Among parous women, breastfeeding duration was the sum of the number of months of reported breastfeeding for each child. Among postmenopausal women, oral HT use was based on self-reported use of estrogen and progesterone pills for purposes other than birth control and classified as never-use (<1 month use), current use (≥1 month use and last use up to 6 months ago), recent use (≥1 month use and last use from 6 months up to 2 years ago), and past use (≥1 month use and last use at least 2 years ago). Family history of breast cancer was based on female first-degree relatives with breast cancer. Age at menarche, highest level of education, and number of alcoholic drinks per week were also based on self-report.

BMI (in kilograms per square meter) was calculated from height and weight measurements and included in models as a categorical variable (<25 kg/m^2^, 25 to <30 kg/m^2^, and ≥30 kg/m^2^). Time of blood collection was included in models by using 2-hour categories. Time since last menstrual period was the number of days between blood draw and the participant's most recent menstrual period and was used as a continuous variable.

### Statistical analysis

Odds ratios (ORs), 95% confidence intervals (CIs), and Wald *P *values for the association between genotypes and breast cancer were estimated by using unconditional logistic regression, adjusting for study site and age. The more common allele was used to determine the reference group. Per-allele ORs and *P *values for trend were estimated by using a log-additive (one degree of freedom) statistical model. Genotype-specific ORs were estimated using the co-dominant (two degrees of freedom) model.

Haplotype associations for breast cancer risk were explored by using Haplostats [23,24]. For each gene, a score test was used to generate a global *P *value for the overall association between haplotypes and breast cancer by using the sliding window approach with a three-SNP window. ORs, 95% CIs, and *P *values were estimated for individual haplotypes with a frequency of 5% or greater within regions where haplotypes were globally associated with breast cancer (score test global *P *< 0.05). Haplotype effects were modeled as log-additive by using logistic regression and were adjusted for age and study site.

Serum prolactin levels were log-transformed, and linear regression was used to estimate geometric mean prolactin levels by genotype in controls and to estimate the association between genotypes or haplotypes and serum prolactin levels. Previous studies have shown that prolactin levels are higher in premenopausal compared with postmenopausal women and that prolactin levels vary with the day of menstrual cycle in premenopausal women [25,26]. Owing to the differences in average prolactin levels and cycling patterns, we presented data on mean prolactin levels by genotype stratified by menopausal status. Models were adjusted for age, time of blood collection, and time since last menstrual period (premenopausal women only). Additional models that adjusted for factors previously determined to be associated with prolactin levels in this study population - parity in premenopausal women and oral HT use and BMI in postmenopausal women - were constructed [15]. Associations between SNPs and breast cancer risk are shown stratified by menopausal status for direct comparison with mean prolactin levels by genotype and are also presented for all women combined.

Statistical tests were two-sided (alpha = 0.05). Unadjusted *P *values are presented in all tables. We also evaluated the false discovery rate (FDR) by using the Benjamini and Hochberg method [27] to assess the robustness of associations after accounting for multiple comparisons. Statistical analyses were performed by using SAS version 9.1 (SAS Institute Inc., Cary, NC, USA) and R version 2.10.

## Results

### Participant characteristics

The mean age of participants was 56 years, and 73% of cases and 69% of controls were postmenopausal. The majority of cases and controls were parous (cases 86%, controls 89%), reported having breastfed (cases 79%, controls 81%), and never drank alcohol (cases 65%, controls 67%) (Table 1). Most postmenopausal participants reported that they had never used oral HT (cases 82%, controls 88%) (Table 1). Cases reported a family history of breast cancer more often than controls (cases 10%, controls 6%) (Table 1).

### Association between *PRL *and *PRLR *SNPs and breast cancer risk

We examined the association between variant alleles in 25 SNPs - 7 *PRL *and 18 *PRLR *- and breast cancer risk. *PRLR *SNPs rs7718468 and rs13436213 were associated with postmenopausal breast cancer risk (rs7718468 per-allele OR 1.16, 95% CI 1.03 to 1.30; rs13436213 per-allele OR 1.13, 95% CI 1.01 to 1.26). For both SNPs, the association was strongest for women carrying the minor allele homozygous genotype (Table 3). One SNP was associated with premenopausal breast cancer: *PRLR *rs249537 (per-allele OR 1.39, 95% CI 1.07 to 1.82).

**Table 3:** *PRLR *single-nucleotide polymorphisms associated with breast cancer in the Polish Breast Cancer Study, by menopausal status^a^

Single-nucleotide polymorphism	Case	Control	OR (95% CI)^b^	*P*	Mean prolactin in sampled controls^c^, ng/mL (95% CI)	*P *trend
All subjects						
*PRLR *rs249537						
CC	1,331	1,540	Referent			
CT	388	400	1.12 (0.96-1.32)	0.15		
TT	19	10	2.19 (1.02-4.73)	0.05		
T vs. C			1.17 (1.01-1.35)	0.04		
*PRLR *rs13436213						
GG	698	829	Referent			
AG	814	885	1.09 (0.95-1.26)	0.21		
AA	237	238	1.18 (0.96-1.46)	0.11		
A vs. G			1.09 (0.99-1.20)	0.08		
*PRLR *rs7718468						
TT	739	895	Referent			
CT	771	831	1.12 (0.98-1.29)	0.10		
CC	214	209	1.24 (1.00-1.54)	0.05		
C vs. T			1.12 (1.01-1.23)	0.02		
						
Premenopausal						
*PRLR *rs249537						
CC	351	492	Referent		5.50 (4.05-7.47)	
CT	104	122	1.19 (0.88-1.59)	0.26	5.51 (3.86-7.87)	
TT	12	2	8.42 (1.89-37.9)	0.01	3.83 (1.36-10.82)	
T vs. C			1.39 (1.07-1.80)	0.01		0.84
*PRLR *rs13436213						
GG	196	269	Referent		5.35 (3.90-7.33)	
AG	220	265	1.14 (0.88-1.48)	0.32	5.30 (3.83-7.32)	
AA	55	82	0.92 (0.62-1.36)	0.67	5.40 (3.75-7.80)	
A vs. G			1.01 (0.85-1.20)	0.92		0.98
*PRLR *rs7718468						
TT	204	286	Referent		5.79 (4.22-7.94)	
CT	209	250	1.18 (0.91-1.53)	0.21	5.46 (3.90-7.64)	
CC	50	72	0.97 (0.65-1.46)	0.90	5.21 (3.6-7.54)	
C vs. T			1.05 (0.87-1.25)	0.63		0.31
						
Postmenopausal						
*PRLR *rs249537						
CC	980	1,048	Referent		6.36 (5.01-8.07)	
CT	284	278	1.11 (0.92-1.34)	0.28	6.45 (4.99-8.33)	
TT	7	8	0.94 (0.34-2.61)	0.90	6.42 (3.64-11.32)	
T vs. C			1.09 (0.92-1.31)	0.33		0.81
*PRLR *rs13436213						
GG	502	560	Referent		6.29 (4.97-7.95)	
AG	594	620	1.07 (0.91-1.27)	0.40	6.52 (5.15-8.25)	
AA	182	156	1.32 (1.03-1.68)	0.03	6.48 (5.01-8.39)	
A vs. G			1.13 (1.01-1.26)	0.04		0.48
*PRLR *rs7718468						
TT	535	609	Referent		6.47 (5.09-8.22)	
CT	562	581	1.11 (0.94-1.31)	0.21	6.86 (5.39-8.74)	
CC	164	137	1.39 (1.07-1.79)	0.01	6.41 (4.89-8.4)	
C vs. T			1.16 (1.03-1.30)	0.01		0.56

^a^A complete list of single-nucleotide polymorphism associations with breast cancer risk can be found in Supplementary table S2 in Additional file 1. ^b^Odds ratio (OR) and confidence interval (CI) adjusted for age and study site. ^c^Adjusted for age, study site, time of blood collection, and time since last period (premenopausal only). *PRLR*, prolactin receptor (gene).

### Association between *PRL *and *PRLR *SNPs and serum prolactin levels in controls

**Figure 1:**
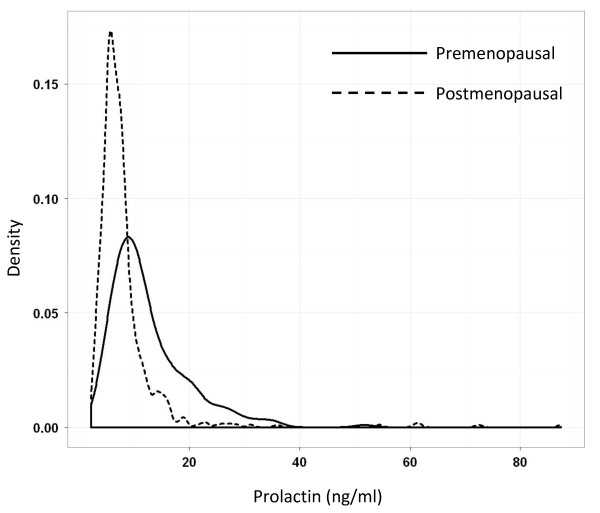
**The distribution of serum prolactin concentrations in controls, by menopausal status**. Prolactin concentrations in Polish Breast Cancer Study control subjects ranged from 2.1 to 348.4 ng/mL, and the majority were less than 50 ng/mL. The unadjusted geometric mean (interquartile range) prolactin concentrations were 10.89 ng/mL (7.80 to 15.30) in premenopausal controls and 6.99 ng/mL (5.30 to 8.60) in postmenopausal controls.

Serum prolactin was measured in 773 controls, and levels were higher in premenopausal women compared with postmenopausal women (Figure 1). Several SNPs in *PRLR *were significantly associated with prolactin levels in premenopausal controls: rs62355518 (*P *= 0.01), rs10941235 (*P *= 0.01), rs1610218 (*P *= 0.01), rs34024951 (*P *= 0.02), and rs9292575 (*P *= 0.03) (Table 4). Results were similar after adjustment for additional factors related to prolactin levels in controls (Supplementary table S3a in Additional file 1).

**Table 4:** Single-nucleotide polymorphisms associated with serum prolactin levels in the Polish Breast Cancer Study^a^

Single-nucleotide polymorphism	Number	Geometric mean^b^	95% CI	*P *trend
Premenopausal controls				
*PRLR*				
rs62355518				
AA	140	11.16	8.81-14.15	0.01
AG	55	8.90	6.87-11.51	
GG	5	10.61	6.60-17.05	
rs10941235				
GG	101	11.05	8.66-14.08	0.01
AG	90	9.24	7.21-11.85	
AA	12	9.37	6.57-13.37	
rs1610218				
GG	192	10.47	8.26-13.28	0.01
AG	13	6.95	4.81-10.03	
AA	0			
rs34024951				
GG	170	10.25	8.07-13.02	0.02
AG	34	8.38	6.27-11.19	
AA	1	6.60	2.53-17.21	
rs9292575				
CC	162	10.07	7.97-12.71	0.03
AC	39	13.38	10.10-17.72	
AA	3	7.32	4.19-12.80	
Postmenopausal controls				
*PRL*				
rs849872				
AA	300	7.63	6.23-9.35	0.01
AG	126	8.34	6.76-10.28	
GG	13	9.69	7.10-13.24	

^a^A complete list of single-nucleotide polymorphism associations with serum prolactin concentrations can be found in Supplementary tables S3a and S3b in Additional file 1. ^b^Adjusted for age, study site, time of blood collection, and time since last period (premenopausal only). CI, confidence interval; *PRL*, prolactin (gene); *PRLR*, prolactin receptor (gene).

In postmenopausal controls, the G allele of *PRL *rs849872 was associated with higher serum prolactin levels (*P *= 0.01) (Table 4). Closer examination of the prolactin distributions showed that there were several outlier values among postmenopausal controls (Figure 1), and so we performed a sensitivity analysis to determine the effect of these outliers on the association between rs849872 and prolactin in postmenopausal controls; the association was no longer significant after excluding the seven prolactin values greater than 30 ng/mL (*P *= 0.09). In addition, visual examination of the data shows that the distribution of prolactin concentrations was similar by genotype (Supplementary figure S1 in Additional file 2).

### Association between *PRL *and *PRLR *haplotypes, serum prolactin, and breast cancer risk

A sliding window approach was used to identify global haplotype associations with breast cancer. An association was detected for a region encompassing *PRLR *SNPs rs873456, rs7718468, rs34024951, and rs9292575 and postmenopausal breast cancer. Five haplotypes in this region had estimated frequencies of greater than 0.05, and haplotype G-C-G-C was positively associated with breast cancer risk when compared with the referent haplotype G-T-G-C (per-copy OR 1.17, 95% CI 1.02 to 1.34) (Supplementary table S1 in Additional file 1). These haplotypes were not associated with serum prolactin levels in postmenopausal controls (data not shown). There was no association between *PRLR *haplotypes and premenopausal breast cancer or between *PRL *haplotypes and premenopausal or postmenopausal breast cancer.

We used the FDR method to evaluate the robustness of SNP associations with serum prolactin levels and breast cancer risk. When all 25 SNPs tested were accounted for, FDR-adjusted *P *values for SNPs with a nominal *P *value of less than 0.05 ranged from 0.10 to 0.69.

## Discussion

We examined the association between SNPs in *PRL *and *PRLR *and breast cancer risk in a population of Polish women. *PRLR *SNPs rs7718468 and rs13436213 and a haplotype including *PRLR *SNPs rs873456, rs7718468, rs34024951, and rs9292575 were associated with breast cancer in postmenopausal women; however, the SNP associations were not statistically significant after adjustment for multiple comparisons. *PRLR *rs249537 was associated with breast cancer risk in premenopausal women; however, the minor allele homozygote genotype for rs249537 was rare (<1% in controls) and this association requires replication.

Few studies have investigated the association between SNPs in *PRL *and *PRLR *and breast cancer risk. Lee and colleagues [19] evaluated the association between *PRL *and *PRLR *tag SNPs and breast cancer risk in a case-control analysis of approximately 3,500 multiethnic premenopausal and postmenopausal women and reported that *PRL *rs9466314 was associated with increased breast cancer risk and that *PRLR *rs34024951 was associated with decreased risk. rs34024951 was not associated with premenopausal or postmenopausal breast cancer in our study population but, along with rs7718468, was a member of a four-SNP haplotype that was associated with postmenopausal breast cancer risk. This suggests that the region marked by rs34024951 and rs7718468 may be in LD with a breast cancer risk-associated locus. Neither rs34024951 nor rs7718468 or the *PRLR *haplotype was associated with serum prolactin levels in postmenopausal controls in our study; thus, our data do not support the hypothesis that the risk mechanism is linked to circulating prolactin levels.

Replication of the association between rs9466314 and breast cancer risk was limited by population differences in risk allele frequencies. The population studied by Lee and colleagues [19] included multiple ethnic groups, and the rs9466314 risk allele was somewhat common (5% in controls) among African-Americans only; in whites, the risk allele was extremely uncommon (<0.25% in controls). Similarly, rs9466314 was monomorphic in our study, and we were unable to examine the association. Interestingly, the risk allele frequencies for both rs9466314 and rs34024951 were higher in African-Americans compared with other ethnic groups in the analysis by Lee and colleagues, suggesting that there may be a greater chance of discovery of *PRL *and *PRLR *SNPs related to breast cancer risk in African-American or African populations.

Comparison with other studies was also limited by a lack of overlapping SNPs between the studies. None of the SNPs we found to be associated with breast cancer (rs249537, rs7718468, and rs13436213) was included in the analysis by Lee and colleagues [19]. SNPs in LD with rs249537 and 13436213 were not associated with breast cancer in the analysis by Lee and colleagues. No nearby SNPs were in LD with rs7718468 in the HapMap CEU population; therefore, we were unable to check the associations of correlated SNPs in other studies.

In another study, Vaclavicek and colleagues [20] reported that *PRL *promoter SNPs rs1341239 and rs12210179 were positively associated with familial breast cancer. SNP rs12202764, which is in LD with rs12210179, was not associated with breast cancer in our study. SNP rs1341239 was initially selected for genotyping but failed assay design; SNPs in LD with rs1341239 were not genotyped in our study. A positive nonsignificant association between *PRL *rs2244502 and familial breast cancer was also reported in the same study [20], but rs2244502 was not associated with breast cancer in our study.

*In vitro *studies have described polymorphisms in *PRLR *- I76V (rs16871473) and I146L (rs72478580) - [17,18,28] that result in constitutive activation of the prolactin receptor. Three small studies suggest that these polymorphisms may be more prevalent among women with benign breast disease, a breast cancer risk factor. The prevalence rates of I146L were 6% among white women with benign breast disease and 0% among non-white women with benign breast disease and healthy women [17,28]. The prevalence rates of the I76V variant allele were 7% in whites and 22% in non-whites with benign breast disease and 4% in a group of healthy women [18]. To our knowledge, there are no data relating these variants to serum prolactin levels or breast cancer risk. I146L and I76V polymorphisms were not genotyped in the Polish Breast Cancer Study, but these loci may represent appealing targets for future research into *PRLR *genetic variation and breast cancer risk.

Our data did not support the hypothesis that serum prolactin levels differ for women with *PRL *and *PRLR *genotypes associated with breast cancer; prolactin levels did not vary by genotype for any of the breast cancer-associated SNPs in this study (rs13436213, rs249537, and rs7718468). To our knowledge, only Lee and colleagues [19] have examined serum prolactin levels in conjunction with *PRL *and *PRLR *genotypes and breast cancer risk, but examination of serum prolactin levels was restricted to a sample of postmenopausal women only. Nonetheless, in that study, prolactin levels varied by genotype for risk-associated SNP rs9466314, but not rs34024951 [19].

It is likely that SNP-related variations in prolactin do not translate directly into altered disease risk and that other biological factors are at play. Several breast cancer risk factors have been reported to be associated with prolactin levels [29] (reviewed in [14]), and estrogen has been shown to stimulate the *PRL *extrapituitary promoter in breast cancer cell lines [30]. Furthermore, prolactin acts through both autocrine and endocrine pathways (reviewed in [2]). Refinement of tissue-level prolactin and prolactin receptor expression assays may provide a more accurate estimate of the prolactin levels surrounding breast epithelial cells from autocrine and endocrine sources.

Interpretation of our results was limited by the lack of a validation population and by lack of overlap between SNPs genotyped in our and previous *PRL *and *PRLR *SNP studies. Thus, confirmation in other populations is necessary. The results from Bogorad and colleagues [17] and Lee and colleagues [19] suggest that study of rare variants may show more promise than evaluation of common variants only. Collaboration and pooled analyses may be the best ways to analyze alleles with a prevalence of less than 5% with sufficient statistical power. Additionally, SNP rs7735260 had low concordance, indicating that genotype calls for this SNP may not be reliable. However, the high concordance rates among the subset of samples that were genotyped in duplicate indicate that genotyping was generally consistent for other SNPs in the study.

A major strength of this study was the measurement of serum prolactin levels. This allowed us to examine relationships between circulating prolactin levels and risk alleles, addressing the hypothesis that risk alleles have a measurable effect on circulating prolactin levels. Additionally, we conducted this analysis within a large, population-based case-control study. The range of prolactin levels in controls is representative of prolactin concentrations among women in the general Polish population. As discussed above, prolactin concentrations in the breast may differ from circulating concentrations. Measurement of prolactin in breast tissue may provide additional information about the link between *PRL *and *PRLR *genetics and breast cancer risk.

## Conclusions

We found limited evidence for a relationship between common genetic variants in *PRL *or *PRLR *and breast cancer. Future studies should be conducted in ethnically diverse populations and should have sufficient sample size to study rare variants.

## Abbreviations

BMI: body mass index; CEU: Utah residents with Northern and Western European ancestry from the CEPH (Centre d'Etude du Polymorphisme Humain) collection; CI: confidence interval; FDR: false discovery rate; HT: hormone therapy; HWE: Hardy-Weinberg equilibrium; LD: linkage disequilibrium; MAF: minor allele frequency; OR: odds ratio; *PRL*: prolactin (gene); *PRLR*: prolactin receptor (gene); SNP: single-nucleotide polymorphism.

## Competing interests

The authors declare that they have no competing interests.

## Authors' contributions

SJN carried out the analysis and drafted the manuscript. JMF-B and MES contributed to the organization and design of the study and contributed to manuscript preparation. MMG, JL, LAB, BP, BKV, and MG-C contributed to the organization and design of the study. RTF contributed to the data analysis and study design. AAA contributed to the data analysis. RMP contributed to the data analysis and manuscript preparation. SC supervised genotyping. JDF directed the organization and design of the study, data analysis, and manuscript preparation. All authors read and approved the final manuscript.

## Supplementary Material

Additional file 1 **Supplementary tables S1-S3**. Supplementary table S1. *PRLR *haplotypes associated with postmenopausal breast cancer in the Polish Breast Cancer Study. Supplementary table S2. Association between *PRLR *and *PRL *SNPs and breast cancer risk in the Polish Breast Cancer Study. Supplementary table S3a. Association between *PRLR *and *PRL *SNPs and serum prolactin levels among premenopausal controls in the Polish Breast Cancer Study. Supplementary table S3b. Association between *PRLR *and *PRL *SNPs and serum prolactin levels among postmenopausal controls in the Polish Breast Cancer Study. Click here for file

Additional file 2 **Supplementary figure S1**. Log-prolactin distribution in postmenopausal controls, by rs849872 genotype. Click here for file
