# Supplementary material for: Genetic variation in PRL and PRLR, and relationships with serum prolactin levels and breast cancer risk: results from a population-based case-control study in Poland
Source: Breast Cancer Res. 2011 Apr 6;13(2):R42. doi: 10.1186/bcr2864 (PMC3219205; doi:10.1186/bcr2864)
Supplement: Additional file 1 — Supplementary tables S1-S3. Supplementary table S1. PRLR haplotypes associated with postmenopausal breast cancer in the Polish Breast Cancer Study. Supplementary table S2. Association between PRLR and PRL SNPs and breast cancer risk in the Polish Breast Cancer Study. Supplementary table S3a. Association between PRLR and PRL SNPs and serum prolactin levels among premenopausal controls in the Polish Breast Cancer Study. Supplementary table S3b. Association between PRLR and PRL SNPs and serum prolactin levels among postmenopausal controls in the Polish Breast Cancer Study. [file bcr2864-S1.DOC]

Supplementary table 1. *PRLR* haplotypes associated with postmenopausal breast cancer in the Polish Breast Cancer Study.

| SNPs: rs873456, rs7718468, rs34024951, and rs9292575 | | | | | |
| --- | --- | --- | --- | --- | --- |
|  |  | | |  |  |
|  | Frequency | | |  |  |
|  | Overall | Case | Control | OR (95% CI) ^1^ | P-value |
| G-T-G-C | 0.32 | 0.31 | 0.33 | Referent |  |
| G-C-G-C | 0.34 | 0.35 | 0.32 | 1.17 (1.02, 1.34) | 0.02 |
| T-T-G-C | 0.15 | 0.15 | 0.16 | 0.98 (0.83, 1.17) | 0.83 |
| T-T-A-C | 0.10 | 0.11 | 0.10 | 1.18 (0.97, 1.43) | 0.11 |
| T-T-G-A | 0.08 | 0.08 | 0.09 | 0.92 (0.75, 1.14) | 0.47 |

^1^OR, CI – odds ratio and confidence interval, per copy of index haplotype compared to the reference haplotype adjusted for age and study site

Supplementary table 2. Association between *PRLR* and *PRL* SNPs and breast cancer risk in the Polish Breast Cancer Study.

|  | All |  |  |  | Premenopausal | |  |  | Postmenopausal | |  |  |
| --- | --- | --- | --- | --- | --- | --- | --- | --- | --- | --- | --- | --- |
|  | Case | Control | OR (95% CI) | P-value | Case | Control | OR (95% CI) | P-value | Case | Control | OR (95% CI) | P-value |
| ***PRLR*** |  |  |  |  |  |  |  |  |  |  |  |  |
| rs37364 |  |  |  |  |  |  |  |  |  |  |  |  |
| AA | 1020 | 1190 | Referent |  | 274 | 372 | Referent |  | 746 | 818 | Referent |  |
| AC | 648 | 660 | 1.15 (1.00, 1.31) | 0.05 | 170 | 212 | 1.09 (0.85, 1.41) | 0.50 | 478 | 448 | 1.18 (1.00, 1.39) | 0.05 |
| CC | 71 | 86 | 0.96 (0.70, 1.33) | 0.82 | 23 | 25 | 1.26 (0.70, 2.27) | 0.44 | 48 | 61 | 0.87 (0.59, 1.29) | 0.49 |
| P-additive | |  |  | 0.19 |  |  |  | 0.34 |  |  |  | 0.29 |
| rs43215 |  |  |  |  |  |  |  |  |  |  |  |  |
| GG | 1232 | 1425 | Referent |  | 329 | 442 | Referent |  | 903 | 983 | Referent |  |
| AG | 431 | 412 | 1.21 (1.04, 1.42) | 0.02 | 109 | 131 | 1.13 (0.84, 1.51) | 0.42 | 322 | 281 | 1.26 (1.05, 1.51) | 0.01 |
| AA | 23 | 29 | 0.92 (0.53, 1.59) | 0.76 | 10 | 7 | 1.94 (0.73, 5.16) | 0.19 | 13 | 22 | 0.63 (0.32, 1.27) | 0.20 |
| P-additive | |  |  | 0.05 |  |  |  | 0.19 |  |  |  | 0.12 |
| rs249537 |  |  |  |  |  |  |  |  |  |  |  |  |
| CC | 1331 | 1540 | Referent |  | 351 | 492 | Referent |  | 980 | 1048 | Referent |  |
| CT | 388 | 400 | 1.12 (0.96, 1.32) | 0.15 | 104 | 122 | 1.19 (0.88, 1.59) | 0.26 | 284 | 278 | 1.11 (0.92, 1.34) | 0.28 |
| TT | 19 | 10 | 2.19 (1.02, 4.73) | 0.05 | 12 | 2 | 8.42 (1.87, 37.9) | 0.01 | 7 | 8 | 0.94 (0.34, 2.61) | 0.90 |
| P-additive | |  |  | 0.04 |  |  |  | 0.01 |  |  |  | 0.33 |
| rs7734558 | |  |  |  |  |  |  |  |  |  |  |  |
| CC | 431 | 481 | Referent |  | 122 | 148 | Referent |  | 309 | 333 | Referent |  |
| CT | 870 | 1003 | 0.97 (0.83, 1.13) | 0.69 | 231 | 303 | 0.93 (0.69, 1.25) | 0.63 | 639 | 700 | 0.99 (0.82, 1.19) | 0.91 |
| TT | 378 | 421 | 1.00 (0.83, 1.21) | 0.98 | 90 | 138 | 0.80 (0.56, 1.15) | 0.22 | 288 | 283 | 1.10 (0.88, 1.38) | 0.42 |
| P-additive | |  |  | 1.00 |  |  |  | 0.23 |  |  |  | 0.43 |
| rs62355518 | |  |  |  |  |  |  |  |  |  |  |  |
| AA | 1202 | 1369 | Referent |  | 328 | 428 | Referent |  | 874 | 941 | Referent |  |
| AG | 451 | 513 | 1.00 (0.86, 1.16) | 0.98 | 120 | 170 | 0.92 (0.70, 1.21) | 0.56 | 331 | 343 | 1.04 (0.87, 1.24) | 0.66 |
| GG | 52 | 53 | 1.12 (0.76, 1.65) | 0.57 | 10 | 13 | 1.00 (0.43, 2.32) | 1.00 | 42 | 40 | 1.12 (0.72, 1.74) | 0.62 |
| P-additive | |  |  | 0.74 |  |  |  | 0.63 |  |  |  | 0.54 |
| rs10941235 | |  |  |  |  |  |  |  |  |  |  |  |
| GG | 923 | 1038 | Referent |  | 243 | 327 | Referent |  | 680 | 711 | Referent |  |
| AG | 690 | 783 | 0.99 (0.87, 1.13) | 0.89 | 192 | 247 | 1.05 (0.81, 1.35) | 0.72 | 498 | 536 | 0.97 (0.83, 1.14) | 0.72 |
| AA | 131 | 136 | 1.08 (0.84, 1.40) | 0.53 | 34 | 38 | 1.21 (0.74, 1.98) | 0.44 | 97 | 98 | 1.02 (0.76, 1.38) | 0.87 |
| P-additive | |  |  | 0.74 |  |  |  | 0.47 |  |  |  | 0.92 |
| rs13436213 | |  |  |  |  |  |  |  |  |  |  |  |
| GG | 698 | 829 | Referent |  | 196 | 269 | Referent |  | 502 | 560 | Referent |  |
| AG | 814 | 885 | 1.09 (0.95, 1.26) | 0.21 | 220 | 265 | 1.14 (0.88, 1.48) | 0.32 | 594 | 620 | 1.07 (0.91, 1.27) | 0.40 |
| AA | 237 | 238 | 1.18 (0.96, 1.46) | 0.11 | 55 | 82 | 0.92 (0.62, 1.36) | 0.67 | 182 | 156 | 1.32 (1.03, 1.68) | 0.03 |
| P-additive | |  |  | 0.08 |  |  |  | 0.92 |  |  |  | 0.04 |
| rs4425481 | |  |  |  |  |  |  |  |  |  |  |  |
| GG | 1680 | 1861 | Referent |  | 436 | 552 | Referent |  | 1244 | 1309 | Referent |  |
| GT | 180 | 206 | 0.97 (0.78, 1.19) | 0.76 | 53 | 71 | 0.95 (0.65, 1.39) | 0.80 | 127 | 135 | 1.00 (0.77, 1.29) | 0.98 |
| TT | 5 | 10 | 0.56 (0.19, 1.63) | 0.28 | 2 | 2 | 1.18 (0.16, 8.44) | 0.87 | 3 | 8 | 0.40 (0.11, 1.52) | 0.18 |
| P-additive | |  |  | 0.50 |  |  |  | 0.85 |  |  |  | 0.58 |
| rs1610218 | |  |  |  |  |  |  |  |  |  |  |  |
| GG | 1618 | 1825 | Referent |  | 447 | 575 | Referent |  | 1171 | 1250 | Referent |  |
| AG | 143 | 145 | 1.12 (0.88, 1.42) | 0.37 | 31 | 45 | 0.89 (0.55, 1.43) | 0.62 | 112 | 100 | 1.19 (0.90, 1.58) | 0.23 |
| AA | 4 | 4 | 1.14 (0.29, 4.59) | 0.85 |  |  |  |  | 4 | 4 | 1.05 (0.26, 4.22) | 0.95 |
| P-additive | |  |  | 0.37 |  |  |  |  |  |  |  | 0.25 |
| rs17249539 | |  |  |  |  |  |  |  |  |  |  |  |
| CC | 1350 | 1531 | Referent |  | 358 | 488 | Referent |  | 992 | 1043 | Referent |  |
| CG | 367 | 403 | 1.03 (0.88, 1.21) | 0.69 | 99 | 119 | 1.14 (0.84, 1.53) | 0.40 | 268 | 284 | 0.99 (0.82, 1.19) | 0.88 |
| GG | 25 | 24 | 1.18 (0.67, 2.08) | 0.56 | 10 | 7 | 1.98 (0.74, 5.25) | 0.17 | 15 | 17 | 0.94 (0.46, 1.89) | 0.85 |
| P-additive | |  |  | 0.55 |  |  |  | 0.17 |  |  |  | 0.83 |
| rs873456 |  |  |  |  |  |  |  |  |  |  |  |  |
| GG | 767 | 831 | Referent |  | 204 | 266 | Referent |  | 563 | 565 | Referent |  |
| GT | 748 | 869 | 0.93 (0.81, 1.07) | 0.32 | 208 | 275 | 0.99 (0.76, 1.28) | 0.92 | 540 | 594 | 0.91 (0.77, 1.07) | 0.26 |
| TT | 195 | 224 | 0.94 (0.76, 1.17) | 0.60 | 50 | 63 | 1.04 (0.69, 1.58) | 0.85 | 145 | 161 | 0.89 (0.69, 1.15) | 0.39 |
| P-additive | |  |  | 0.40 |  |  |  | 0.93 |  |  |  | 0.25 |
| rs7718468 | |  |  |  |  |  |  |  |  |  |  |  |
| TT | 739 | 895 | Referent |  | 204 | 286 | Referent |  | 535 | 609 | Referent |  |
| CT | 771 | 831 | 1.12 (0.98, 1.29) | 0.10 | 209 | 250 | 1.18 (0.91, 1.53) | 0.21 | 562 | 581 | 1.11 (0.94, 1.31) | 0.21 |
| CC | 214 | 209 | 1.24 (1.00, 1.54) | 0.05 | 50 | 72 | 0.97 (0.65, 1.46) | 0.90 | 164 | 137 | 1.39 (1.07, 1.79) | 0.01 |
| P-additive | |  |  | 0.02 |  |  |  | 0.63 |  |  |  | 0.01 |
| rs34024951 | |  |  |  |  |  |  |  |  |  |  |  |
| GG | 1409 | 1601 | Referent |  | 393 | 505 | Referent |  | 1016 | 1096 | Referent |  |
| AG | 309 | 341 | 1.03 (0.87, 1.22) | 0.73 | 75 | 106 | 0.91 (0.66, 1.26) | 0.56 | 234 | 235 | 1.07 (0.88, 1.31) | 0.50 |
| AA | 22 | 19 | 1.32 (0.71, 2.45) | 0.38 | 3 | 7 | 0.56 (0.14, 2.19) | 0.40 | 19 | 12 | 1.64 (0.79, 3.41) | 0.18 |
| P-additive | |  |  | 0.49 |  |  |  | 0.38 |  |  |  | 0.23 |
| rs9292575 | |  |  |  |  |  |  |  |  |  |  |  |
| CC | 1474 | 1607 | Referent |  | 395 | 499 | Referent |  | 1079 | 1108 | Referent |  |
| AC | 256 | 323 | 0.86 (0.72, 1.03) | 0.11 | 69 | 108 | 0.81 (0.58, 1.13) | 0.21 | 187 | 215 | 0.89 (0.72, 1.11) | 0.30 |
| AA | 8 | 14 | 0.62 (0.26, 1.48) | 0.28 | 3 | 5 | 0.73 (0.17, 3.10) | 0.67 | 5 | 9 | 0.56 (0.19, 1.67) | 0.30 |
| P-additive | |  |  | 0.06 |  |  |  | 0.19 |  |  |  | 0.18 |
| rs10038062 | |  |  |  |  |  |  |  |  |  |  |  |
| AA | 1507 | 1634 | Referent |  | 405 | 501 | Referent |  | 1102 | 1133 | Referent |  |
| AG | 257 | 318 | 0.87 (0.73, 1.05) | 0.14 | 71 | 111 | 0.79 (0.57, 1.09) | 0.16 | 186 | 207 | 0.92 (0.74, 1.14) | 0.44 |
| GG | 12 | 18 | 0.73 (0.35, 1.52) | 0.40 | 0 | 4 |  |  | 12 | 14 | 0.86 (0.40, 1.88) | 0.71 |
| P-additive | |  |  | 0.10 |  |  |  |  |  |  |  | 0.40 |
| rs931741 |  |  |  |  |  |  |  |  |  |  |  |  |
| CC | 850 | 906 | Referent |  | 226 | 278 | Referent |  | 624 | 628 | Referent |  |
| CG | 699 | 820 | 0.91 (0.79, 1.04) | 0.17 | 187 | 273 | 0.84 (0.65, 1.09) | 0.19 | 512 | 547 | 0.94 (0.80, 1.11) | 0.45 |
| GG | 169 | 192 | 0.94 (0.75, 1.18) | 0.58 | 41 | 52 | 0.98 (0.63, 1.53) | 0.94 | 128 | 140 | 0.92 (0.70, 1.19) | 0.51 |
| P-additive | |  |  | 0.27 |  |  |  | 0.43 |  |  |  | 0.39 |
| rs7735260 | |  |  |  |  |  |  |  |  |  |  |  |
| CC | 1133 | 1286 | Referent |  | 313 | 396 | Referent |  | 820 | 890 | Referent |  |
| CT | 396 | 438 | 1.03 (0.88, 1.20) | 0.75 | 100 | 147 | 0.86 (0.64, 1.15) | 0.30 | 296 | 291 | 1.10 (0.91, 1.32) | 0.34 |
| TT | 70 | 59 | 1.35 (0.95, 1.92) | 0.10 | 21 | 17 | 1.59 (0.82, 3.07) | 0.17 | 49 | 42 | 1.29 (0.84, 1.96) | 0.24 |
| P-additive | |  |  | 0.21 |  |  |  | 0.91 |  |  |  | 0.16 |
| rs10068521 | |  |  |  |  |  |  |  |  |  |  |  |
| CC | 1687 | 1870 | Referent |  | 452 | 583 | Referent |  | 1235 | 1287 | Referent |  |
| CG | 83 | 106 | 0.87 (0.65, 1.17) | 0.35 | 24 | 35 | 0.87 (0.51, 1.49) | 0.62 | 59 | 71 | 0.89 (0.62, 1.26) | 0.50 |
| GG | 1 | 3 | 0.37 (0.04, 3.58) | 0.39 | 0 | 2 |  |  | 1 | 1 | 1.09 (0.07, 17.4) | 0.95 |
| P-additive | |  |  | 0.25 |  |  |  |  |  |  |  | 0.53 |
| ***PRL*** |  |  |  |  |  |  |  |  |  |  |  |  |
| rs849872 |  |  |  |  |  |  |  |  |  |  |  |  |
| AA | 1229 | 1372 | Referent |  | 332 | 435 | Referent |  | 897 | 937 | Referent |  |
| AG | 460 | 516 | 1.00 (0.86, 1.15) | 0.95 | 126 | 157 | 1.05 (0.80, 1.38) | 0.74 | 334 | 359 | 0.97 (0.82, 1.16) | 0.75 |
| GG | 35 | 43 | 0.91 (0.58, 1.43) | 0.68 | 6 | 18 | 0.41 (0.16, 1.05) | 0.06 | 29 | 25 | 1.19 (0.69, 2.05) | 0.53 |
| P-additive | |  |  | 0.80 |  |  |  | 0.49 |  |  |  | 0.97 |
| rs849870 |  |  |  |  |  |  |  |  |  |  |  |  |
| GG | 1299 | 1459 | Referent |  | 352 | 460 | Referent |  | 947 | 999 | Referent |  |
| AG | 407 | 463 | 0.99 (0.85, 1.15) | 0.88 | 111 | 143 | 1.01 (0.76, 1.35) | 0.94 | 296 | 320 | 0.98 (0.81, 1.17) | 0.80 |
| AA | 31 | 34 | 1.02 (0.62, 1.67) | 0.93 | 7 | 13 | 0.67 (0.27, 1.71) | 0.41 | 24 | 21 | 1.17 (0.65, 2.12) | 0.60 |
| P-additive | |  |  | 0.93 |  |  |  | 0.73 |  |  |  | 0.97 |
| rs1205960 | |  |  |  |  |  |  |  |  |  |  |  |
| GG | 968 | 1087 | Referent |  | 263 | 334 | Referent |  | 705 | 753 | Referent |  |
| AG | 670 | 761 | 0.99 (0.86, 1.13) | 0.87 | 174 | 249 | 0.89 (0.69, 1.14) | 0.35 | 496 | 512 | 1.04 (0.88, 1.22) | 0.65 |
| AA | 101 | 111 | 1.02 (0.77, 1.36) | 0.88 | 30 | 33 | 1.16 (0.69, 1.95) | 0.58 | 71 | 78 | 0.96 (0.69, 1.35) | 0.82 |
| P-additive | |  |  | 0.98 |  |  |  | 0.78 |  |  |  | 0.87 |
| rs849886 |  |  |  |  |  |  |  |  |  |  |  |  |
| GG | 503 | 561 | Referent |  | 143 | 181 | Referent |  | 360 | 380 | Referent |  |
| AG | 835 | 957 | 0.97 (0.84, 1.13) | 0.72 | 223 | 313 | 0.90 (0.68, 1.19) | 0.47 | 612 | 644 | 1.00 (0.84, 1.21) | 0.96 |
| AA | 374 | 405 | 1.03 (0.86, 1.24) | 0.75 | 93 | 114 | 1.03 (0.73, 1.47) | 0.86 | 281 | 291 | 1.01 (0.82, 1.26) | 0.90 |
| P-additive | |  |  | 0.81 |  |  |  | 0.98 |  |  |  | 0.90 |
| rs2244502 | |  |  |  |  |  |  |  |  |  |  |  |
| AA | 897 | 997 | Referent |  | 224 | 299 | Referent |  | 673 | 698 | Referent |  |
| AT | 782 | 914 | 0.95 (0.83, 1.08) | 0.45 | 228 | 270 | 1.14 (0.89, 1.46) | 0.31 | 554 | 644 | 0.90 (0.77, 1.05) | 0.17 |
| TT | 193 | 188 | 1.14 (0.92, 1.42) | 0.24 | 45 | 60 | 1.01 (0.66, 1.54) | 0.98 | 148 | 128 | 1.19 (0.92, 1.55) | 0.18 |
| P-additive | |  |  | 0.65 |  |  |  | 0.57 |  |  |  | 0.81 |
| rs12202764 | |  |  |  |  |  |  |  |  |  |  |  |
| AA | 943 | 1069 | Referent |  | 247 | 321 | Referent |  | 696 | 748 | Referent |  |
| AT | 661 | 736 | 1.02 (0.89, 1.17) | 0.80 | 178 | 249 | 0.94 (0.73, 1.21) | 0.61 | 483 | 487 | 1.07 (0.91, 1.26) | 0.42 |
| TT | 133 | 149 | 1.01 (0.79, 1.30) | 0.92 | 42 | 47 | 1.16 (0.74, 1.81) | 0.53 | 91 | 102 | 0.97 (0.72, 1.31) | 0.84 |
| P-additive | |  |  | 0.83 |  |  |  | 0.88 |  |  |  | 0.72 |
| rs3756824 | |  |  |  |  |  |  |  |  |  |  |  |
| GG | 1640 | 1825 | Referent |  | 447 | 576 | Referent |  | 1193 | 1249 | Referent |  |
| CG | 75 | 91 | 0.92 (0.67, 1.25) | 0.59 | 20 | 30 | 0.85 (0.47, 1.51) | 0.57 | 55 | 61 | 0.95 (0.66, 1.38) | 0.80 |
| CC | 1 | 4 | 0.28 (0.03, 2.52) | 0.26 |  |  |  |  | 1 | 4 | 0.27 (0.03, 2.46) | 0.25 |
| P-additive | |  |  | 0.36 |  |  |  |  |  |  |  | 0.48 |

^1^odds ratio and 95 % confidence interval, adjusted for age and study site

Supplementary Table 3a. Association between *PRLR* and *PRL* SNPs and serum prolactin levels among premenopausal controls in the Polish Breast Cancer Study.

|  |  |  | **Unadjusted** | |  | **Multivariable model 1^3^** | | | **Multivariable model 2^4^** | |  |
| --- | --- | --- | --- | --- | --- | --- | --- | --- | --- | --- | --- |
| **SNP** | **Genotype** | **N** | **Mean^1^** | **95% CI^2^** | **P-trend** | **Mean^1^** | **95% CI^2^** | **P-trend** | **Mean^1^** | **95% CI^2^** | **P-trend** |
| ***PRLR*** |  |  |  |  |  |  |  |  |  |  |  |
| rs37364 | AA | 123 | 11.14 | 10.16 - 12.23 | 0.55 | 10.24 | 7.95 - 13.20 | 0.49 | 13.73 | 9.91 - 19.02 | 0.65 |
|  | AC | 68 | 11.02 | 9.73 - 12.48 |  | 10.22 | 7.84 - 13.32 |  | 13.90 | 9.87 - 19.56 |  |
|  | CC | 11 | 9.78 | 7.18 - 13.34 |  | 8.77 | 6.06 - 12.68 |  | 12.04 | 7.85 - 18.49 |  |
| rs43215 | GG | 154 | 11.3 | 10.42 - 12.26 | 0.92 | 10.09 | 7.81 - 13.04 | 0.46 | 13.35 | 9.59 - 18.59 | 0.56 |
|  | AG | 36 | 11.49 | 9.71 - 13.60 |  | 9.56 | 7.13 - 12.83 |  | 12.83 | 8.90 - 18.50 |  |
|  | AA | 1 | 10.20 | 3.71 - 28.03 |  | 7.60 | 2.92 - 19.73 |  | 10.31 | 3.92 - 27.10 |  |
| rs249537 | CC | 168 | 10.83 | 10.00 - 11.72 | 0.55 | 10.09 | 7.88 - 12.93 | 0.71 | 13.82 | 10.01 - 19.08 | 0.63 |
|  | CT | 38 | 11.63 | 9.85 - 13.74 |  | 10.02 | 7.44 - 13.50 |  | 13.60 | 9.51 - 19.44 |  |
|  | TT | 1 | 8.80 | 3.15 - 24.58 |  | 6.32 | 2.39 - 16.73 |  | 8.70 | 3.28 - 23.10 |  |
| rs7734558 | CC | 55 | 12.01 | 10.48 - 13.76 | 0.21 | 9.89 | 7.55 - 12.95 | 0.27 | 13.53 | 9.62 - 19.03 | 0.25 |
|  | CT | 88 | 10.81 | 9.71 - 12.04 |  | 10.33 | 7.97 - 13.40 |  | 13.89 | 10.04 - 19.23 |  |
|  | TT | 52 | 10.59 | 9.21 - 12.19 |  | 11.01 | 8.29 - 14.62 |  | 15.08 | 10.61 - 21.44 |  |
| rs62355518 | AA | 140 | 11.66 | 10.71 - 12.7 | 0.07 | 11.16 | 8.81 - 14.15 | 0.01 | 15.24 | 11.18 - 20.78 | 0.01 |
|  | AG | 55 | 9.61 | 8.39 - 11.01 |  | 8.90 | 6.87 - 11.51 |  | 12.12 | 8.76 - 16.76 |  |
|  | GG | 5 | 12.24 | 7.80 - 19.20 |  | 10.61 | 6.60 - 17.05 |  | 13.73 | 8.37 - 22.52 |  |
| rs10941235 | GG | 101 | 11.77 | 10.65 - 13.01 | 0.13 | 11.05 | 8.66 - 14.08 | 0.01 | 15.06 | 10.97 - 20.66 | 0.01 |
|  | AG | 90 | 10.65 | 9.58 - 11.84 |  | 9.24 | 7.21 - 11.85 |  | 12.56 | 9.13 - 17.27 |  |
|  | AA | 12 | 10.02 | 7.50 - 13.40 |  | 9.37 | 6.57 - 13.37 |  | 12.70 | 8.48 - 19.02 |  |
| rs13436213 | GG | 91 | 10.95 | 9.84 - 12.19 | 0.81 | 10.15 | 7.89 - 13.06 | 0.87 | 13.64 | 9.75 - 19.09 | 0.72 |
|  | AG | 88 | 10.82 | 9.70 - 12.06 |  | 9.74 | 7.61 - 12.47 |  | 13.06 | 9.38 - 18.18 |  |
|  | AA | 25 | 11.49 | 9.37 - 14.10 |  | 10.76 | 8.04 - 14.40 |  | 14.90 | 10.19 - 21.80 |  |
| rs4425481 | GG | 176 | 10.97 | 10.16 - 11.84 | 0.33 | 10.67 | 8.5 - 13.38 | 0.20 | 13.88 | 10.15 - 18.99 | 0.11 |
|  | GT | 25 | 9.54 | 7.79 - 11.69 |  | 9.32 | 6.92 - 12.56 |  | 11.97 | 8.36 - 17.14 |  |
|  | TT | 2 | 11.87 | 5.79 - 24.34 |  | 9.76 | 4.97 - 19.15 |  | 11.30 | 5.75 - 22.20 |  |
| rs1610218 | GG | 192 | 11.14 | 10.34 – 12.00 | 0.08 | 10.47 | 8.26 - 13.28 | 0.01 | 14.02 | 10.21 - 19.24 | 0.01 |
|  | AG | 13 | 8.59 | 6.46 - 11.41 |  | 6.95 | 4.81 - 10.03 |  | 9.52 | 6.20 - 14.60 |  |
|  | AA | 0 |  |  |  |  |  |  |  |  |  |
| rs17249539 | CC | 158 | 11.10 | 10.23 - 12.03 | 0.98 | 10.26 | 8.04 - 13.08 | 0.83 | 13.83 | 10.05 - 19.02 | 0.77 |
|  | CG | 44 | 10.74 | 9.21 - 12.51 |  | 10.25 | 7.89 - 13.32 |  | 13.88 | 9.93 - 19.40 |  |
|  | GG | 2 | 15.48 | 7.56 - 31.73 |  | 14.72 | 5.91 - 36.68 |  | 20.73 | 8.20 - 52.41 |  |
| rs873456 | GG | 83 | 11.01 | 9.85 - 12.31 | 0.65 | 9.98 | 7.78 - 12.81 | 0.96 | 13.52 | 9.79 - 18.68 | 0.82 |
|  | GT | 99 | 10.94 | 9.88 - 12.12 |  | 9.98 | 7.75 - 12.85 |  | 13.70 | 9.83 - 19.09 |  |
|  | TT | 21 | 11.99 | 9.61 - 14.96 |  | 10.07 | 7.30 - 13.89 |  | 13.82 | 9.41 - 20.30 |  |
| rs7718468 | TT | 96 | 11.43 | 10.31 - 12.67 | 0.58 | 10.83 | 8.41 - 13.94 | 0.18 | 15.22 | 10.92 - 21.21 | 0.17 |
|  | CT | 82 | 11.06 | 9.89 - 12.37 |  | 9.78 | 7.60 - 12.60 |  | 13.30 | 9.68 - 18.29 |  |
|  | CC | 23 | 10.80 | 8.75 - 13.34 |  | 9.74 | 7.25 - 13.08 |  | 14.04 | 9.66 - 20.39 |  |
| rs34024951 | GG | 170 | 11.20 | 10.35 - 12.12 | 0.19 | 10.25 | 8.07 - 13.02 | 0.02 | 13.90 | 10.15 - 19.04 | 0.02 |
|  | AG | 34 | 10.20 | 8.55 - 12.16 |  | 8.38 | 6.27 - 11.19 |  | 11.39 | 8.00 - 16.23 |  |
|  | AA | 1 | 5.80 | 2.08 - 16.19 |  | 6.60 | 2.53 - 17.21 |  | 9.17 | 3.49 - 24.07 |  |
| rs9292575 | CC | 162 | 10.44 | 9.65 - 11.29 | 0.02 | 10.07 | 7.97 - 12.71 | 0.03 | 13.8 | 10.15 - 18.75 | 0.02 |
|  | AC | 39 | 14.11 | 12.02 - 16.56 |  | 13.38 | 10.1 - 17.72 |  | 18.49 | 13.07 - 26.14 |  |
|  | AA | 3 | 7.91 | 4.44 - 14.10 |  | 7.32 | 4.19 - 12.80 |  | 10.39 | 5.75 - 18.79 |  |
| rs10038062 | AA | 165 | 10.91 | 10.08 - 11.81 | 0.69 | 10.16 | 7.98 - 12.95 | 0.80 | 13.87 | 10.08 - 19.07 | 0.81 |
|  | AG | 40 | 11.32 | 9.63 - 13.30 |  | 9.96 | 7.59 - 13.05 |  | 13.60 | 9.67 - 19.12 |  |
|  | GG | 0 |  |  |  |  |  |  |  |  |  |
| rs931741 | CC | 89 | 10.93 | 9.82 - 12.17 | 0.59 | 10.28 | 7.99 - 13.23 | 0.94 | 13.47 | 9.69 - 18.73 | 0.93 |
|  | CG | 100 | 10.87 | 9.82 - 12.03 | 0.98 | 10.05 | 7.84 - 12.88 | 0.83 | 13.37 | 9.57 - 18.68 |  |
|  | GG | 15 | 12.44 | 9.57 - 16.16 |  | 10.56 | 7.46 - 14.96 |  | 13.94 | 9.27 - 20.98 |  |
| rs7735260 | CC | 133 | 11.36 | 10.41 - 12.39 |  | 10.16 | 7.92 - 13.04 |  | 13.04 | 9.27 - 18.34 | 0.11 |
|  | CT | 46 | 9.44 | 8.14 - 10.94 |  | 8.45 | 6.23 - 11.46 |  | 11.13 | 7.46 - 16.58 |  |
|  | TT | 6 | 9.92 | 6.59 - 14.93 |  | 9.09 | 5.60 - 14.74 |  | 12.01 | 6.95 - 20.74 |  |
| rs10068521 | CC | 191 | 11.04 | 10.26 - 11.89 | 0.97 | 10.38 | 8.19 - 13.17 | 0.53 | 14.13 | 10.33 - 19.33 | 0.54 |
|  | CG | 14 | 9.87 | 7.52 - 12.95 |  | 10.56 | 7.31 - 15.26 |  | 14.26 | 9.42 - 21.58 |  |
|  | GG | 1 | 24.50 | 8.86 - 67.77 |  | 16.78 | 6.48 - 43.42 |  | 23.07 | 8.87 - 60.04 |  |
| ***PRL*** |  |  |  |  |  |  |  |  |  |  |  |
| rs849872 | AA | 147 | 10.88 | 10.01 - 11.81 | 0.39 | 10.18 | 8.02 - 12.93 | 0.32 | 13.63 | 9.95 - 18.66 | 0.34 |
|  | AG | 51 | 12.11 | 10.53 - 13.94 |  | 11.36 | 8.72 - 14.81 |  | 15.05 | 10.82 - 20.94 |  |
|  | GG | 6 | 10.35 | 6.88 - 15.58 |  | 9.67 | 6.11 - 15.31 |  | 13.15 | 7.96 - 21.73 |  |
| rs849870 | GG | 154 | 11.02 | 10.16 - 11.95 | 0.90 | 10.43 | 8.19 - 13.27 | 0.77 | 14.11 | 10.27 - 19.40 | 0.84 |
|  | AG | 46 | 11.76 | 10.15 - 13.64 |  | 10.96 | 8.38 - 14.33 |  | 14.62 | 10.49 - 20.39 |  |
|  | AA | 5 | 9.00 | 5.75 - 14.09 |  | 9.75 | 6.13 - 15.51 |  | 13.41 | 8.07 - 22.27 |  |
| rs1205960 | GG | 102 | 11.21 | 10.14 - 12.40 | 0.62 | 10.46 | 8.17 - 13.38 | 0.50 | 14.04 | 10.20 - 19.33 | 0.70 |
|  | AG | 87 | 10.96 | 9.83 - 12.23 |  | 9.84 | 7.65 - 12.67 |  | 13.43 | 9.64 - 18.70 |  |
|  | AA | 17 | 10.50 | 8.20 - 13.45 |  | 10.12 | 7.19 - 14.25 |  | 14.01 | 9.32 - 21.06 |  |
| rs849886 | GG | 64 | 11.59 | 10.21 - 13.16 | 0.65 | 10.99 | 8.35 - 14.45 | 0.39 | 15.03 | 10.62 - 21.26 | 0.37 |
|  | AG | 112 | 10.73 | 9.75 - 11.81 |  | 10.06 | 7.91 - 12.80 |  | 13.76 | 9.97 – 19.00 |  |
|  | AA | 27 | 11.38 | 9.36 - 13.84 |  | 10.31 | 7.58 - 14.02 |  | 14.05 | 9.69 - 20.36 |  |
| rs2244502 | AA | 91 | 10.74 | 9.67 - 11.92 | 0.50 | 10.32 | 8.11 - 13.13 | 0.41 | 13.02 | 9.44 - 17.96 | 0.48 |
|  | AT | 87 | 10.89 | 9.78 - 12.12 |  | 10.41 | 8.24 - 13.14 |  | 13.17 | 9.58 - 18.10 |  |
|  | TT | 28 | 11.68 | 9.68 - 14.11 |  | 11.45 | 8.60 - 15.24 |  | 14.17 | 10.03 - 20.03 |  |
| rs12202764 | AA | 99 | 11.36 | 10.25 - 12.59 | 0.62 | 10.29 | 7.98 - 13.26 | 0.96 | 14.24 | 10.16 - 19.95 | 0.62 |
|  | AT | 98 | 10.52 | 9.49 - 11.67 |  | 9.99 | 7.81 - 12.77 |  | 13.58 | 9.83 - 18.77 |  |
|  | TT | 10 | 12.21 | 8.83 - 16.88 |  | 11.44 | 7.74 - 16.89 |  | 14.23 | 9.43 - 21.49 |  |
| rs3756824 | GG | 187 | 10.92 | 10.14 - 11.76 | 0.33 | 9.96 | 7.83 - 12.68 | 0.63 | 13.39 | 9.68 - 18.53 | 0.51 |
|  | CG | 14 | 12.55 | 9.57 - 16.46 |  | 10.66 | 7.47 - 15.20 |  | 14.66 | 9.60 - 22.38 |  |
|  | CC | 0 |  |  |  |  |  |  |  |  |  |

^1^mean – geometric mean

^2^CI – confidence interval

^3^adjusted for age, study site, time since last period, time of blood collection

^4^adjusted for age, study site, time since last period, time of blood collection, parity

Supplementary Table 3b. Association between *PRLR* and *PRL* SNPs and serum prolactin levels among postmenopausal controls in the Polish Breast Cancer Study.

|  |  |  | **Unadjusted** | |  | **Multivariable model 1^3^** | | | **Multivariable model 2^4^** | |  |
| --- | --- | --- | --- | --- | --- | --- | --- | --- | --- | --- | --- |
| **SNP** | **Genotype** | **N** | **Mean^1^** | **95% CI^2^** | **P-trend** | **Mean^1^** | **95% CI^2^** | **P-trend** | **Mean^1^** | **95% CI^2^** | **P-trend** |
| ***PRLR*** |  |  |  |  |  |  |  |  |  |  |  |
| rs37364 | AA | 271 | 6.83 | 6.46 - 7.22 | 0.19 | 7.70 | 6.20 - 9.55 | 0.20 | 9.87 | 6.66 - 14.62 | 0.14 |
|  | AC | 149 | 7.08 | 6.57 - 7.63 |  | 7.91 | 6.35 - 9.85 |  | 10.30 | 6.94 - 15.28 |  |
|  | CC | 21 | 7.78 | 6.37 - 9.50 |  | 8.89 | 6.67 - 11.85 |  | 11.40 | 7.44 - 17.47 |  |
| rs43215 | GG | 332 | 6.90 | 6.58 - 7.23 | 0.94 | 8.14 | 6.65 - 9.96 | 0.85 | 8.80 | 5.98 - 12.96 | 0.97 |
|  | AG | 95 | 6.88 | 6.30 - 7.52 |  | 8.05 | 6.48 - 9.99 |  | 8.76 | 5.89 - 13.03 |  |
|  | AA | 3 | 6.79 | 4.13 - 11.18 |  | 8.25 | 4.91 - 13.85 |  | 9.15 | 4.97 - 16.84 |  |
| rs249537 | CC | 360 | 6.90 | 6.58 - 7.23 | 0.71 | 7.76 | 6.28 - 9.59 | 0.85 | 9.60 | 6.61 - 13.95 | 0.92 |
|  | CT | 82 | 7.07 | 6.40 - 7.81 |  | 7.84 | 6.24 - 9.84 |  | 9.58 | 6.53 - 14.04 |  |
|  | TT | 3 | 6.76 | 4.01 - 11.39 |  | 7.89 | 4.54 - 13.72 |  | 9.15 | 5.10 - 16.42 |  |
| rs7734558 | TT | 102 | 6.95 | 6.38 - 7.58 | 0.45 | 7.63 | 6.18 - 9.43 | 0.38 | 9.90 | 6.78 - 14.45 | 0.27 |
|  | CT | 243 | 6.98 | 6.60 - 7.38 |  | 8.00 | 6.50 - 9.85 |  | 10.26 | 7.07 - 14.89 |  |
|  | CC | 102 | 6.63 | 6.09 - 7.23 |  | 8.05 | 6.48 - 10.01 |  | 10.61 | 7.28 - 15.46 |  |
| rs62355518 | AA | 311 | 6.79 | 6.45 - 7.15 | 0.37 | 7.73 | 6.26 - 9.56 | 0.46 | 10.11 | 6.68 - 15.31 | 0.52 |
|  | AG | 119 | 7.52 | 6.92 - 8.16 |  | 8.40 | 6.73 - 10.48 |  | 10.97 | 7.24 - 16.62 |  |
|  | GG | 15 | 5.94 | 4.71 - 7.50 |  | 6.93 | 5.05 - 9.51 |  | 9.02 | 5.59 - 14.55 |  |
| rs10941235 | GG | 234 | 6.77 | 6.38 - 7.18 | 0.59 | 7.62 | 6.16 - 9.42 | 0.59 | 9.15 | 6.32 - 13.24 | 0.56 |
|  | AG | 186 | 7.33 | 6.86 - 7.83 |  | 8.20 | 6.64 - 10.14 |  | 9.80 | 6.79 - 14.13 |  |
|  | AA | 30 | 6.25 | 5.30 - 7.37 |  | 7.11 | 5.48 - 9.23 |  | 8.74 | 5.84 - 13.07 |  |
| rs13436213 | GG | 201 | 6.78 | 6.37 - 7.21 | 0.46 | 7.87 | 6.39 - 9.68 | 0.81 | 9.52 | 6.65 - 13.61 | 0.71 |
|  | AG | 199 | 7.04 | 6.61 - 7.49 |  | 8.06 | 6.54 - 9.93 |  | 9.78 | 6.82 - 14.03 |  |
|  | AA | 48 | 7.00 | 6.17 - 7.95 |  | 7.85 | 6.24 - 9.87 |  | 9.59 | 6.63 - 13.86 |  |
| rs4425481 | GG | 446 | 6.91 | 6.63 - 7.21 | 0.48 | 7.45 | 6.10 - 9.09 | 0.37 | 10.62 | 7.53 - 14.99 | 0.50 |
|  | GT | 49 | 6.84 | 6.02 - 7.77 |  | 7.46 | 5.88 - 9.46 |  | 10.39 | 7.27 - 14.83 |  |
|  | TT | 3 | 11.50 | 6.86 - 19.27 |  | 12.23 | 7.09 - 21.11 |  | 18.12 | 9.86 - 33.30 |  |
| rs1610218 | GG | 421 | 6.93 | 6.63 - 7.24 | 0.72 | 7.76 | 6.31 - 9.54 | 0.66 | 10.25 | 7.00 - 15.03 | 0.50 |
|  | AG | 34 | 7.55 | 6.47 - 8.81 |  | 8.46 | 6.59 - 10.88 |  | 11.30 | 7.48 - 17.05 |  |
|  | AA | 2 | 4.53 | 2.40 - 8.57 |  | 5.29 | 2.77 - 10.10 |  | 7.75 | 3.77 - 15.93 |  |
| rs17249539 | CC | 355 | 7.03 | 6.70 - 7.38 | 0.33 | 7.80 | 6.32 - 9.63 | 0.54 | 10.28 | 6.99 - 15.13 | 0.52 |
|  | CG | 94 | 6.69 | 6.09 - 7.34 |  | 7.62 | 6.10 - 9.52 |  | 9.94 | 6.72 - 14.70 |  |
|  | GG | 4 | 6.53 | 4.15 - 10.27 |  | 6.86 | 4.21 - 11.17 |  | 9.89 | 5.49 - 17.81 |  |
| rs873456 | GG | 196 | 6.89 | 6.46 - 7.35 | 0.56 | 8.21 | 6.61 - 10.19 | 0.52 | 10.32 | 6.98 - 15.28 | 0.73 |
|  | GT | 190 | 7.32 | 6.86 - 7.82 |  | 8.72 | 7.01 - 10.85 |  | 11.05 | 7.49 - 16.30 |  |
|  | TT | 52 | 6.17 | 5.44 - 7.00 |  | 7.32 | 5.79 - 9.26 |  | 9.41 | 6.27 - 14.13 |  |
| rs7718468 | TT | 215 | 6.83 | 6.42 - 7.26 | 0.58 | 7.72 | 6.24 - 9.56 | 0.75 | 9.40 | 6.50 - 13.59 | 0.98 |
|  | CT | 186 | 7.23 | 6.77 - 7.73 |  | 8.14 | 6.54 - 10.13 |  | 9.72 | 6.69 - 14.11 |  |
|  | CC | 44 | 6.77 | 5.90 - 7.76 |  | 7.50 | 5.89 - 9.55 |  | 9.06 | 6.14 - 13.39 |  |
| rs34024951 | GG | 365 | 6.89 | 6.57 - 7.23 | 0.57 | 8.11 | 6.55 - 10.03 | 0.73 | 9.92 | 6.84 - 14.39 | 0.59 |
|  | AG | 80 | 7.46 | 6.73 - 8.26 |  | 8.68 | 6.92 - 10.88 |  | 10.71 | 7.28 - 15.76 |  |
|  | AA | 5 | 5.14 | 3.41 - 7.75 |  | 5.88 | 3.73 - 9.27 |  | 7.65 | 4.43 - 13.22 |  |
| rs9292575 | CC | 380 | 6.96 | 6.64 - 7.29 | 0.87 | 7.80 | 6.31 - 9.63 | 1.00 | 10.11 | 6.87 - 14.88 | 0.72 |
|  | AC | 67 | 6.97 | 6.24 - 7.78 |  | 7.72 | 6.09 - 9.79 |  | 10.19 | 6.77 - 15.33 |  |
|  | AA | 1 | 9.20 | 3.72 - 22.75 |  | 10.67 | 4.31 - 26.41 |  | 15.96 | 6.13 - 41.52 |  |
| rs10038062 | AA | 389 | 6.99 | 6.67 - 7.32 | 0.85 | 8.10 | 6.57 - 9.98 | 0.74 | 9.88 | 6.83 - 14.29 | 0.72 |
|  | AG | 64 | 6.86 | 6.12 - 7.68 |  | 8.00 | 6.31 - 10.15 |  | 9.76 | 6.63 - 14.37 |  |
|  | GG | 3 | 7.39 | 4.36 - 12.51 |  | 7.28 | 4.19 - 12.63 |  | 8.82 | 4.69 - 16.58 |  |
| rs931741 | CC | 223 | 6.83 | 6.43 - 7.26 | 0.82 | 7.87 | 6.37 - 9.72 | 0.94 | 9.49 | 6.57 - 13.71 | 1.00 |
|  | CG | 171 | 7.26 | 6.78 - 7.77 |  | 8.47 | 6.82 - 10.52 |  | 10.29 | 7.14 - 14.83 |  |
|  | GG | 45 | 6.25 | 5.46 - 7.14 |  | 7.21 | 5.68 - 9.15 |  | 8.72 | 5.95 - 12.77 |  |
| rs7735260 | CC | 307 | 6.88 | 6.54 - 7.24 | 1.00 | 7.68 | 6.16 - 9.58 | 0.98 | 10.61 | 7.18 - 15.69 | 0.90 |
|  | CT | 93 | 7.11 | 6.48 - 7.80 |  | 7.92 | 6.27 – 10.00 |  | 11.05 | 7.43 - 16.43 |  |
|  | TT | 11 | 6.12 | 4.67 - 8.02 |  | 6.85 | 4.92 - 9.53 |  | 8.97 | 5.77 - 13.95 |  |
| rs10068521 | CC | 436 | 6.88 | 6.60 - 7.18 | 0.99 | 7.85 | 6.43 - 9.58 | 0.91 | 9.51 | 6.56 - 13.78 | 0.96 |
|  | CG | 23 | 6.89 | 5.74 - 8.27 |  | 7.93 | 6.08 - 10.34 |  | 9.47 | 6.33 - 14.15 |  |
|  | GG | 0 |  |  |  |  |  |  |  |  |  |
| ***PRL*** |  |  |  |  |  |  |  |  |  |  |  |
| rs849872 | AA | 300 | 6.78 | 6.45 - 7.13 | 0.04 | 7.63 | 6.23 - 9.35 | 0.01 | 9.88 | 6.85 - 14.26 | 0.01 |
|  | AG | 126 | 7.36 | 6.81 - 7.95 |  | 8.34 | 6.76 - 10.28 |  | 10.78 | 7.44 - 15.62 |  |
|  | GG | 13 | 7.93 | 6.24 - 10.09 |  | 9.69 | 7.10 - 13.24 |  | 12.8 | 8.19 - 20.01 |  |
| rs849870 | GG | 328 | 6.96 | 6.62 - 7.32 | 0.63 | 7.85 | 6.37 - 9.67 | 0.38 | 9.44 | 6.56 - 13.60 | 0.47 |
|  | AG | 110 | 7.10 | 6.52 - 7.74 |  | 8.04 | 6.46 – 10.00 |  | 9.66 | 6.67 – 14.00 |  |
|  | AA | 11 | 7.26 | 5.54 - 9.53 |  | 8.92 | 6.32 - 12.57 |  | 10.45 | 6.57 - 16.62 |  |
| rs1205960 | GG | 251 | 6.94 | 6.56 - 7.35 | 0.79 | 7.73 | 6.26 - 9.55 | 0.49 | 9.49 | 6.59 - 13.66 | 0.45 |
|  | AG | 176 | 6.95 | 6.49 - 7.44 |  | 7.91 | 6.40 - 9.78 |  | 9.74 | 6.72 - 14.10 |  |
|  | AA | 28 | 7.19 | 6.07 - 8.53 |  | 8.14 | 6.22 - 10.64 |  | 10.02 | 6.66 - 15.09 |  |
| rs849886 | GG | 129 | 6.91 | 6.38 - 7.48 | 0.78 | 7.80 | 6.25 - 9.74 | 0.95 | 9.23 | 6.33 - 13.48 | 0.92 |
|  | AG | 220 | 7.01 | 6.60 - 7.46 |  | 7.80 | 6.32 - 9.63 |  | 9.46 | 6.55 - 13.67 |  |
|  | AA | 94 | 7.02 | 6.39 - 7.71 |  | 7.77 | 6.18 - 9.77 |  | 9.14 | 6.25 - 13.38 |  |
| rs2244502 | AA | 244 | 6.95 | 6.56 - 7.37 | 0.86 | 7.62 | 6.22 - 9.34 | 0.75 | 10.72 | 7.68 - 14.95 | 0.90 |
|  | AT | 225 | 6.82 | 6.42 - 7.24 |  | 7.54 | 6.16 - 9.24 |  | 10.45 | 7.49 - 14.60 |  |
|  | TT | 39 | 7.33 | 6.34 - 8.47 |  | 8.04 | 6.34 - 10.19 |  | 11.02 | 7.67 - 15.83 |  |
| rs12202764 | AA | 249 | 6.99 | 6.60 - 7.41 | 0.76 | 8.17 | 6.60 - 10.11 | 0.80 | 10.00 | 6.91 - 14.48 | 0.66 |
|  | AT | 174 | 7.01 | 6.54 - 7.51 |  | 8.20 | 6.61 - 10.18 |  | 10.12 | 6.95 - 14.74 |  |
|  | TT | 26 | 6.63 | 5.54 - 7.94 |  | 7.78 | 5.93 - 10.21 |  | 9.09 | 6.06 - 13.62 |  |
| rs3756824 | GG | 419 | 6.98 | 6.68 - 7.29 | 0.61 | 7.85 | 6.37 - 9.68 | 0.73 | 10.13 | 6.89 - 14.90 | 0.62 |
|  | CG | 19 | 7.63 | 6.20 - 9.39 |  | 8.44 | 6.28 - 11.36 |  | 11.04 | 7.19 - 16.94 |  |
|  | CC | 3 | 6.57 | 3.90 - 11.07 |  | 7.28 | 4.19 - 12.63 |  | 9.29 | 4.49 - 19.22 |  |

^1^mean – geometric mean

^2^CI – confidence interval

^3^adjusted for age, study site, time of blood collection

^4^adjusted for age, study site, time of blood collection, BMI, oral HT use
