# Supplementary figures and images for: Genetic variation in PRL and PRLR, and relationships with serum prolactin levels and breast cancer risk: results from a population-based case-control study in Poland
Source: Breast Cancer Res. 2011 Apr 6;13(2):R42. doi: 10.1186/bcr2864 (PMC3219205; doi:10.1186/bcr2864)

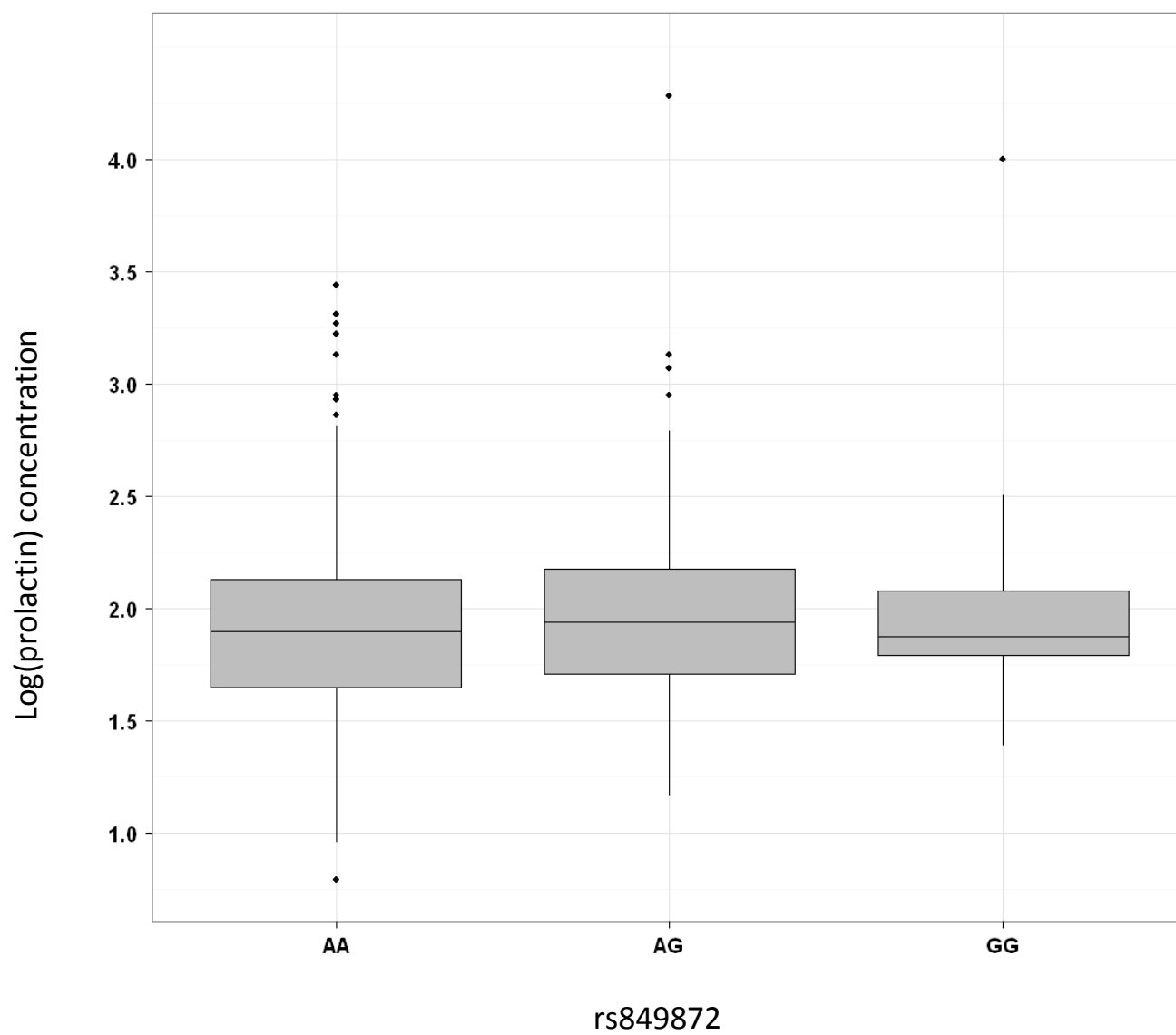

Supplement: Additional file 2 — Supplementary figure S1. Log-prolactin distribution in postmenopausal controls, by rs849872 genotype. [file bcr2864-S2.PDF]
